# Chronic osteomyelitis diagnosis using MRI: optimising radiomics models through multi-level region of interest extensions

**DOI:** 10.3389/fbioe.2026.1773423

**Published:** 2026-07-14

**Authors:** Hao Zheng, Qiyu Jia, Lin Jie, Jiaye Zhang, Shijie Jiang, Abudusalamu Alimujiang, Jian Guo, Junna Wang, Lanqi Fu, Zengru Xie, Chuang Ma

**Affiliations:** 1 Department of Radiology, The First Affiliated Hospital of Zhejiang Chinese Medical University (Zhejiang Provincial Hospital of Chinese Medicine), Hangzhou, China; 2 School of Medicine, Nankai University, Tianjin, China; 3 Department of Orthopedics, The First Affiliated Hospital of Xinjiang Medical University, Urumqi, China; 4 Xinjiang Clinical Research Center for Mental Health, The First Affiliated Hospital of Xinjiang Medical University, Xinjiang, China

**Keywords:** chronic osteomyelitis, machine learning, radiomics, region of interest, surgical decision-making

## Abstract

**Purpose:**

Chronic osteomyelitis is a persistent infectious bone disease characterised by high recurrence, in which surgical debridement plays a crucial role. Conventional debridement mainly depends on surgeons’ clinical experience and visual interpretation of imaging findings, which are inherently subjective and may not accurately define the boundary between infected and healthy tissue. Radiomics demonstrates substantial potential in this domain; however, it faces critical challenges, including region of interest (ROI) selection and spatial mapping. To enhance the diagnostic accuracy of radiomics in osteomyelitis and support preoperative evaluation, this study introduces an innovative multi-level ROI expansion framework. This approach aims to enable comprehensive and precise lesion characterisation as well as to provide a quantitative reference for determining the extent of pathological involvement.

**Patients and Methods:**

Clinical and imaging data from 102 patients with suspected chronic osteomyelitis of the long bones were retrospectively reviewed. The following three ROI models were constructed: an initial ROI model, a 5-mm expansion ROI model and a 10-mm expansion ROI model. Each model underwent radiographic feature analysis and was analysed using four machine learning algorithms: K-nearest neighbour, support vector machine, random forest and logistic regression. Diagnostic performance was assessed using receiver operating characteristic curve analysis, clinical impact curve evaluation and performance metrics including area under the curve (AUC), sensitivity, specificity and accuracy.

**Results:**

Logistic regression algorithm consistently outperformed the other algorithms across all ROI expansion ranges. Compared with the 0-mm and 10-mm ranges, all models demonstrated superior performance within the 5-mm feature range. The DeLong test revealed significant differences among the logistic regression submodels (*p* < 0.05), with the 5-mm expansion model achieving the highest AUC.

**Conclusion:**

The proposed multi-level ROI expansion framework enhances the radiomics-based diagnosis of chronic osteomyelitis. By comparing diagnostic performance across expanded ROI models, we demonstrated that the combination of a 5-mm expanded ROI and logistic regression yielded the highest diagnostic efficacy. These findings indicate that the perilesional region contains critical pathological information, thereby providing theoretical support for the clinical strategy of moderate debridement expansion. Furthermore, the 10-mm expanded ROI model demonstrated distinct advantages in detecting more extensive tissue alterations. Collectively these two ROI expansion strategies offer surgeons a more comprehensive lesion assessment and data-driven insights, thereby facilitating precise surgical planning.

## 1 Introduction

Chronic osteomyelitis is a persistent infectious bone disorder that presents substantial challenges in clinical management. Moreover, surgical debridement remains the most effective treatment strategy for controlling this condition (Ren et al., 2023). However, surgical success requires careful balance; notably, insufficient debridement may leave residual infection and lead to recurrence, whereas overly aggressive debridement may result in unnecessary loss to healthy bone tissue (Masters et al., 2022; Hotchen et al., 2020; Zeitlinger, 2019; Bosse et al., 2002). Traditionally, surgeons determine the extent of debridement based on clinical experience and visual interpretation of imaging findings. This approach is inherently subjective and influenced by individual variability, making it difficult to accurately distinguish infected tissue from healthy bone.

Radiomics, as emerging quantitative imaging analysis approach, has demonstrated transformative potential in this field (Hassani et al., 2019; Lambin et al., 2017; Gillies et al., 2015). By extracting high-dimensional quantitative features from medical images, radiomics provides objective quantitative standards for disease characterisation and has shown empirical value in improving diagnostic accuracy (Bera et al., 2022; Witt et al., 2020; Huang et al., 2016). In our previous study, we applied the support vector machine (SVM) algorithm and demonstrated that a radiomics-based model incorporating a 5-mm expanded region of interest (ROI) outperformed the conventional ROI model in diagnosing chronic osteomyelitis (Jia et al., 2024). However, excessive expansion may adversely affect model performance and compromise diagnostic accuracy. Moreover, although prior studies have primarily focused on diagnostic performance, the effect of ROI selection on defining the pathological extent of disease has not been thoroughly investigated. Importantly, precise alignment between the diagnostic ROI and the actual surgical field remains a significant clinical challenge. An ROI that is too small may overlook critical lesion areas, resulting in incomplete diagnostic information and reduced accuracy. Conversely, an excessively large ROI may include substantial amounts of normal tissue, thus increasing feature heterogeneity and analytical complexity.

Similar considerations apply to the theoretical relationship between diagnostic ROI boundaries and surgical margins. Appropriate expansion is necessary to capture occult pathological changes, whereas excessive expansion may lead to unnecessary tissue damage. Therefore, accurately determining the optimal expansion range is essential for enhancing diagnostic precision and guiding surgical decision-making.

**FIGURE 1:**
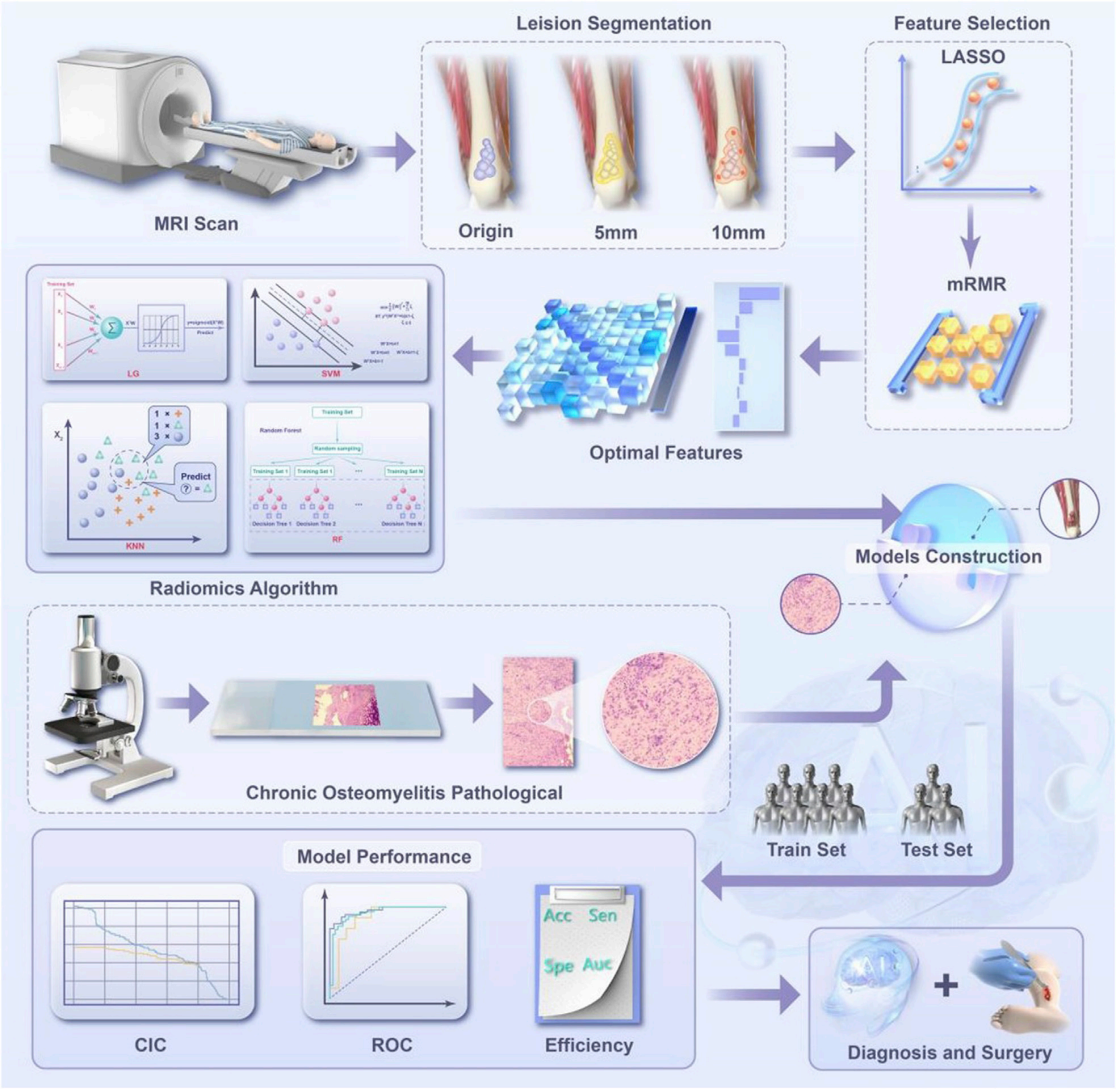
Radiomics-based predictive model construction workflow for chronic osteomyelitis.

To further improve the diagnostic accuracy of radiomics in osteomyelitis and to explore its utility in boundary assessment, this study proposes an innovative multi-level ROI expansion framework. of the following three ROI models were constructed: an initial ROI model, a 5-mm expanded ROI model and a 10-mm expanded ROI model. Each model underwent radiographic feature analysis and was evaluated using four machine learning (ML) algorithms. Our objective was to identify the most appropriate ROI expansion range and optimise the corresponding algorithmic model. This study seeks to establish a more comprehensive and precise lesion assessment strategy, providing a scientific foundation for understanding the spatial distribution of radiomic features for chronic osteomyelitis. We anticipate that this framework will support the development of personalised preoperative planning strategies, facilitating an optimal balance between complete lesion removal and preservation of healthy bone tissue (Figure 1).

## 2 Materials and methods

### 2.1 Data collection

A retrospective analysis was performed on the clinical and imaging data of 102 patients with suspected chronic osteomyelitis of the long bones (Figure 1) who were treated at the First Affiliated Hospital of Xinjiang Medical University between January 2016 and January 2023. The study cohort was predominantly male (n = 69/102, 67.7%), with a mean age of 44 years (range: 19–69 years). Strict inclusion and exclusion criteria were established to ensure diagnostic accuracy and population homogeneity. Eligible patients were those with a high clinical suspicion of chronic osteomyelitis of the long bones requiring surgical intervention. The diagnosis was confirmed by intraoperative histopathological examination, defined as either consistent pathogen isolation from at least two sites or the presence of well-defined sinus tracts directly communicating with to the long bone. Cases involving specific infections, such as mycobacterial infection, were excluded. Medical records included demographic data (sex and age), anatomical site of infection, intraoperative microbiological culture results, treatment strategies, serum biomarkers and MRI findings after admission. Exclusion criteria comprised pregnancy, breastfeeding, presence of metal implants or and diagnoses of acute osteomyelitis, Charcot arthropathy, diabetes mellitus or chronic osteomyelitis located outside the long bones. For patients with multiple hospitalisations, only the medical record most relevant to chronic osteomyelitis of the long bones was considered. Based on retrospective pathological confirmation, 57 patients were with chronic osteomyelitis and 45 with non-chronic osteomyelitis. The study was approved by the Ethics Committee of the First Affiliated Hospital of Xinjiang Medical University, with an exemption from informed consent (K202308-11). All patient data were anonymised and de-identified before analysis to ensure privacy protection.

### 2.2 MR scanning protocol

MRI examinations were performed using a 1.5-T MR scanner (SIEMENS). The scanning parameters were as follows: T_1_-weighted imaging (T_1_WI) with a repetition time (TR) of 600 ms and echo time (TE) = 9.5 ms; T_2_-weighted imaging (T_2_WI) with a TR of 3,000 ms and TE of 88 ms; and fat-suppressed T_2_-weighted imaging (FS T_2_WI) with a TR of 3,600 ms and TE of 83 ms. The field of view was 320 mm, with a matrix size of 256 × 256. All patients underwent routine sequences, including T_1_WI, T_2_WI and T_2_-based short tau inversion recovery (STIR). Imaging planes (coronal, sagittal and axial) were selected according to lesion location. Slice thickness was 4 mm, with an interslice gap of 0.4 mm.

#### 2.2.1 Image selection

MRI data were retrieved from the Picture Archiving and Communication System and reviewed by a radiologist with over 10 years of experience in musculoskeletal imaging. Image quality was verified to ensure complete sequences and absence of artifacts. Diagnostic criteria included low signal intensity on T1WI and high signal intensity on T2WI and STIR sequences in areas consistent with bone marrow inflammation.

#### 2.2.2 Lesion and perilesional area delineation

**FIGURE 2:**
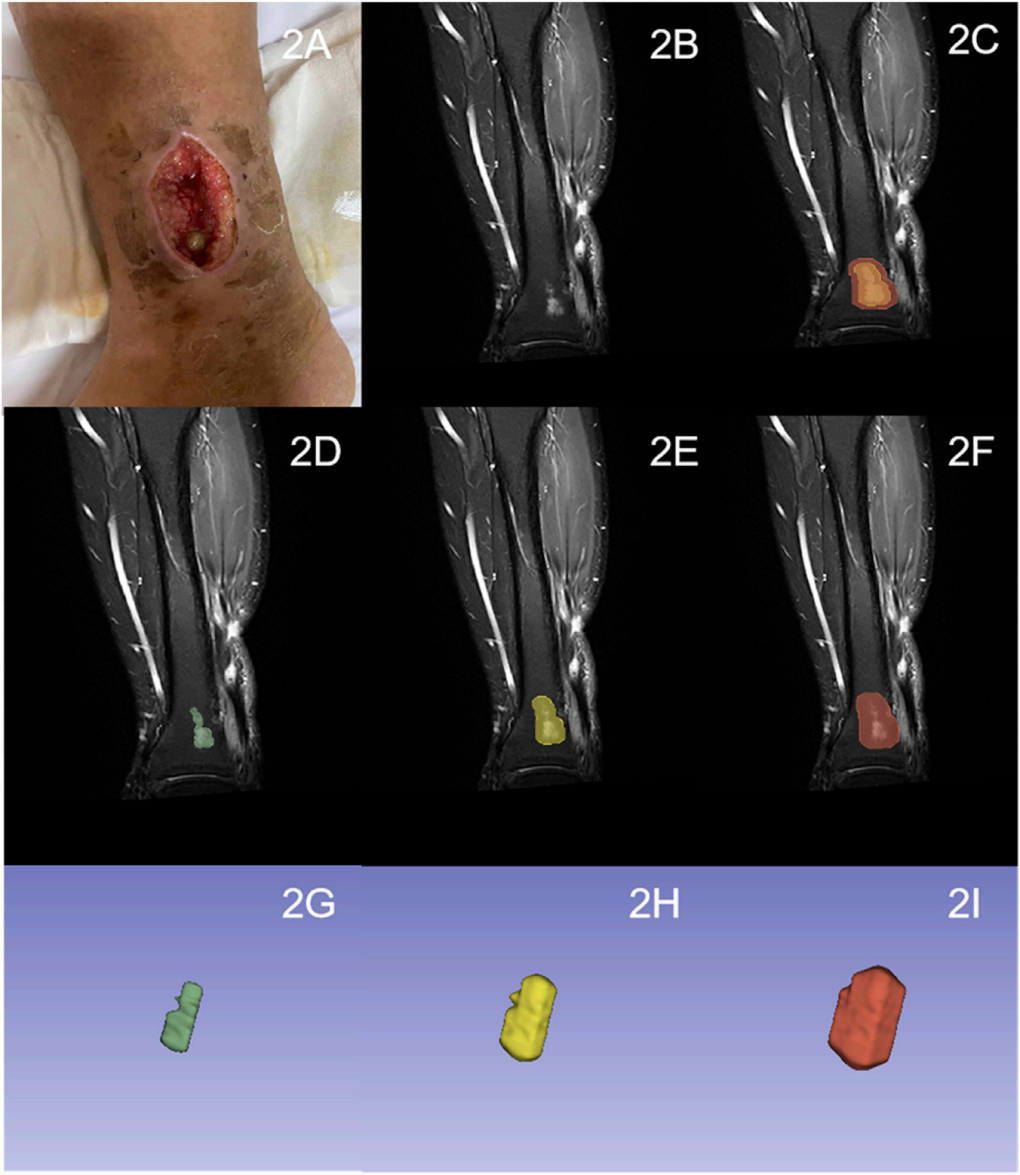
Osteomyelitis outlined using 3D Slicer. **(A)** Macroscopic photo of a patient with chronic osteomyelitis. **(B)** Osteomyelitis in the lateral-distal tibia on the coronal view of the STIR sequence. **(C)** Comparison of ROI for the three types of osteomyelitis. **(D)** Osteomyelitis outlined within the original ROI. **(E)** Osteomyelitis outlined within 5-mm expanded ROI. **(F)** Osteomyelitis outlined within 10-mm expanded ROI. **(G)** 3D model of osteomyelitis reconstructed from the original ROI. **(H)** 3D model of osteomyelitis reconstructed from 5-mm expanded ROI. **(I)** 3D model of osteomyelitis reconstructed from 10-mm expanded ROI.

(1) Lesion delineation was performed using 3D Slicer (version 5.3.0) software, simultaneously outlining the outer contour and bounding box of the lesion. The ROI was delineated in the coronal plane using the STIR sequence based on the MRI T2 sequence as a reference. Subsequently, semi-automatic delineation was applied to the original ROI, showing the lesion on the image as well as the lesion area expanded by 5 and 10 mm (expanded ROI) from the original ROI. Next, manual adjustments were made to confirm the delineation scope and prevent any extension of the delineation beyond the bone structure. (2) Notably, manual delineation was conducted on each lesion plane, carefully avoiding areas of necrosis and haemorrhage while ensuring comprehensive coverage of the lesion substance. (3) Furthermore, for lesions with unclear borders, distinct high signal areas were delineated. (4) In cases with multiple lesions, only the largest lesion was delineated. Lesion and perilesional area delineation results have been illustrated in Figure 2. (5) Two radiologists, each with over a decade of expertise in musculoskeletal imaging diagnostics, conducted the ROI delineation and review.

#### 2.2.3 Feature extraction

Radiomic features were extracted using the pyradiomics module integrated into 3D Slicer. All MR images were resampled to an isotropic voxel size of 1.0 × 1.0 × 1.0 mm via trilinear interpolation. Intensity normalisation was performed using Z-score standardisation, and grey-level discretisation was conducted with a fixed bin width of 25. A total of 1,037 multi-dimensional radiomic features were extracted from each ROI. These features cover shape features; first-order statistical features and higher-order texture features derived from grey-level co-occurrence matrix (GLCM), grey-level size zone matrix, grey-level dependence matrix, grey-level run length matrix and neighbourhood grey-level dependence matrix.

Wavelet and Laplacian of Gaussian (LoG) filters were applied to enhance feature extraction. Wavelet decomposition enabled multi-scale analysis by iteratively separating image components at different frequency levels. However, LoG filtering primarily enhanced edge information by emphasising regions with substantial grey-level variation. The sigma parameter controlled the scale of LoG filtering: smaller sigma values enhanced fine texture details, whereas larger values emphasised broader structural patterns (van Griethuysen et al., 2017; Lambin et al., 2012).

#### 2.2.4 Statistical analysis

All analyses were performed using Python (version 3.9.13). Radiomic features were processed separately for the original ROI, 5-mm expanded ROI and 10-mm expanded ROI.

##### 2.2.4.1 Data preprocessing and cohort splitting

Radiomic features were standardised using Z-score normalisation. Patients were then randomly divided into a training cohort (70%) and a test cohort (30%) via stratified sampling based on histopathological outcomes. Feature selection procedures were conducted exclusively in the training cohort, whereas the independent test cohort was reserved for final model evaluation.

##### 2.2.4.2 Segmentation reproducibility

To evaluate segmentation reproducibility, 30 randomly selected cases were independently segmented by two radiologists (over 10 years of experience). Interobserver agreement was assessed using the intraclass correlation coefficient (ICC) (two-way random-effects model). Features with ICC ≥0.75 were considered reproducible and retained.

##### 2.2.4.3 Feature selection

Dimensionality reduction was performed using the independent samples t-test, least absolute shrinkage and selection operator (LASSO) regression, and maximum relevance–minimum redundancy (mRMR) analysis.

##### 2.2.4.4 Model construction and internal validation

The following 4 ML algorithms were applied: K-nearest neighbour (KNN), SVM, random forest (RF) and logistic regression (LR). Hyperparameter configurations are presented in Table 1.

**TABLE 1:** Hyperparameters selected for each model.

Model	Settings
KNN	n_neighbors = 5
SVM	C = 1.303, gamma = 0.139
RF	n_estimators = 1,000, max_depth = 15, min_samples_split = 8
Logistic regression	max_iter = 10,000, random_state = 50

The predictive models were constructed for ROI type. Hyperparameters were optimised using GridSearchCV. A 5-fold cross-validation in the training cohort was used as an internal validation strategy to reduce overfitting and obtain more robust performance estimates (Simon et al., 2011).

##### 2.2.4.5 Model performance evaluation

Histopathological findings served as the reference standard. Diagnostic performance was assessed using receiver operating characteristic (ROC) curve analysis and clinical impact curve (CIC) assessment. Performance metrics included area under the curve (AUC), sensitivity, specificity and accuracy. The DeLong test was applied to compare differences between ROC curves. Calibration performance was evaluated using calibration curves and quantified using the Brier score, with lower scores indicating better calibration. A two-sided *p-*value of <0.05 was considered statistically significant.

## 3 Results

### 3.1 Reproducibility analysis

Interobserver reproducibility analysis of osteomyelitis lesions and expanded ROIs delineated by the two radiologists demonstrated strong agreement. All ICCs exceeded 0.75, indicating satisfactory segmentation reliability and feature stability.

### 3.2 Radiomics feature extraction

Radiomic features were extracted using the pyradiomics module integrated into 3D Slicer from the following three ROI configurations: the original ROI, the 5-mm expanded ROI and the 10-mm expanded ROI.

**FIGURE 3:**
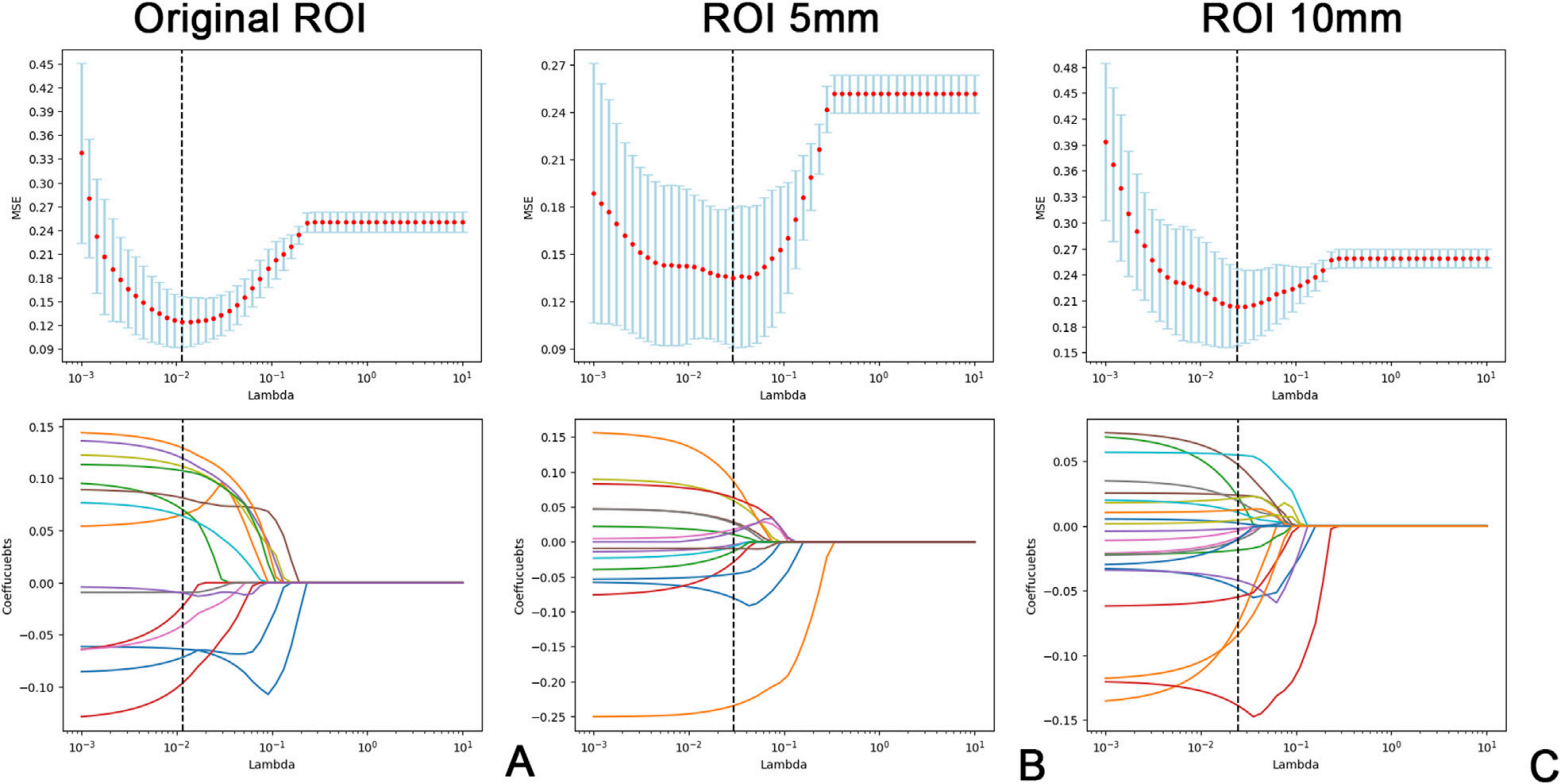
Texture feature selection using t-tests and selection operator (LASSO) for radiomics. **(A)** Original ROI texture feature selection. **(B)** 5-mm expanded ROI texture feature selection. **(C)** 10-mm expanded ROI texture feature selection.

**FIGURE 4:**
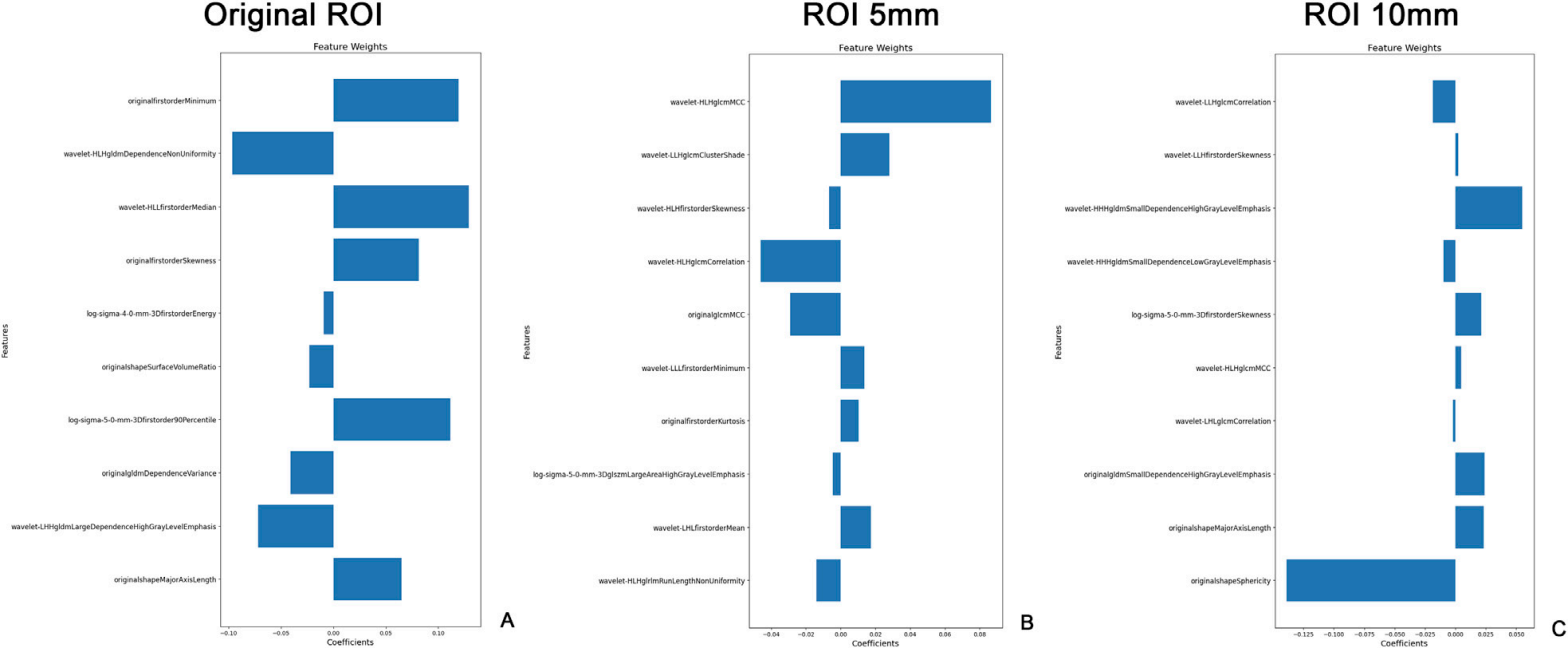
Features weight selected for the original and expanded ROIs. **(A)** Features weight within the original ROI. **(B)** Features weight within the 5-mm expanded ROI. **(C)** Features weight within the 10-mm expanded ROI.

From the initial set of 1,037 features, the independent samples t-test and LASSO regression were applied for preliminary feature selection. A total of 82, 70 and 226 features were retained for the original, 5-mm expanded and 10-mm expanded ROIs, respectively. The effectiveness of the LASSO method in selecting lesion-related texture features is illustrated in Figure 3. To further reduce dimensionality and enhance model efficiency, mRMR analysis was performed. This approach identifies feature subsets with maximal correlation to the target outcome (maximum relevance) while minimising interfeature redundancy (minimum redundancy). The top 10 most representative features were selected from each ROI configuration. Figure 4 presents these selected features along with their corresponding weights across the three datasets.

**FIGURE 5:**
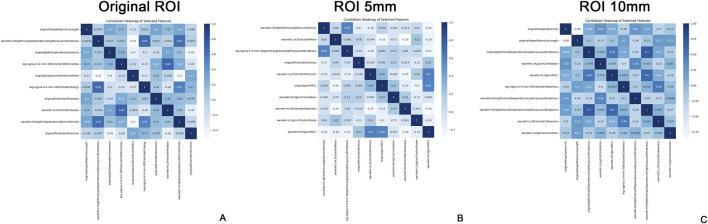
Correlation matrix heatmap. **(A)** Original ROI correlation matrix heat map. **(B)** 5-mm expanded ROI correlation matrix heat map. **(C)** 10-mm expanded ROI correlation matrix heat map.

Moreover, a correlation matrix heat map (Figure 5) was generated to visualise interfeature relationships within each dataset. All correlation coefficients were <0.7, indicating the absence of significant multi-collinearity and confirming the independence of the selected features.

### 3.3 Comparison of model performance

As summarised in Table 2, the diagnostic performance of 4 ML algorithms—KNN, RF, SVM and LR—was evaluated using radiomic features derived from the three ROI extension ranges (0, 5 and 10 mm). Performance metrics included AUC, accuracy (Acc), specificity (Spec) and sensitivity (Sen). Across all expansion ranges, LR consistently achieved the highest AUC, accuracy and sensitivity compared with the other algorithms.

**TABLE 2:** Comparison of diagnostic performance across different ROI and algorithm models.

Extension (mm)	Model	AUC	Acc	Spec	Sen
0	KNN	0.820 (0.710–0.910)	0.790	0.714	0.839
	RF	0.780 (0.350–0.920)	0.741	0.769	0.750
	SVM	0.700 (0.61–0.900)	0.774	0.656	0.842
	Logistic regression	0.890 (0.860–0.940)	0.804	0.719	0.889
5	KNN	0.830 (0.700–0.930)	0.822	0.805	0.788
	RF	0.840 (0.720–0.910)	0.785	0.683	0.867
	SVM	0.780 (0.730–0.900)	0.806	0.805	0.788
	Logistic regression	0.910 (0.870–1.00)	0.851	0.875	0.909
10	KNN	0.730 (0.600–0.850)	0.750	0.842	0.724
	RF	0.740 (0.680–0.800)	0.691	0.618	0.732
	SVM	0.800 (0.780–0.910)	0.720	0.712	0.749
	Logistic regression	0.850 (0.760–0.940)	0.764	0.750	0.800

**FIGURE 6:**
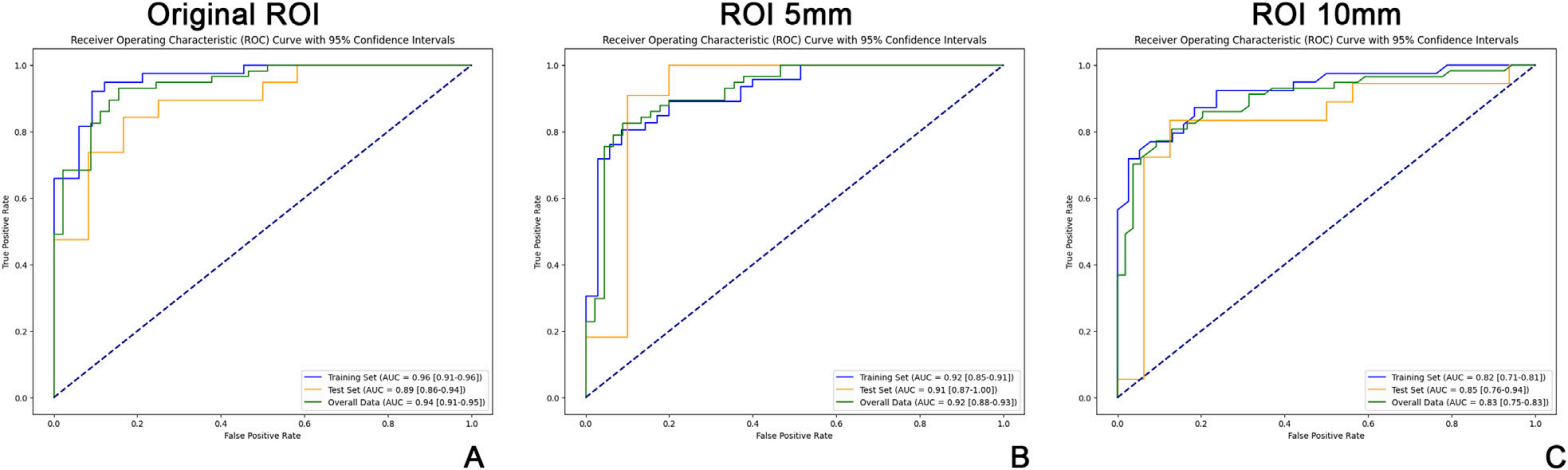
Comparison of the model performance. **(A)** AUC of the original ROI. **(B)** AUC of the 5-mm expanded ROI. **(C)** AUC of the 10-mm expanded ROI.

Among the three ROI configurations, models based on the 5-mm expansion demonstrated superior performance, with higher AUC, accuracy, specificity and sensitivity than those based on the 0 mm or 10 mm expansions. In contrast, extending the ROI to 10 mm resulted in a decline in performance across all metrics. The DeLong test revealed statistically significant differences (*p* < 0.05) among the LR models constructed with the three extension ranges (0, 5 and 10 mm), as shown in the ROC curves in Figure 6. Notably, the 5-mm expansion model achieved the highest AUC. These findings suggest that moderate perilesional expansion enhances diagnostic performance, whereas excessive expansion may introduce redundant or confounding information, thereby impairing accuracy. This emphasizes the importance of selecting an optimal ROI expansion range in radiomics-based diagnosis of osteomyelitis.

**FIGURE 7:**
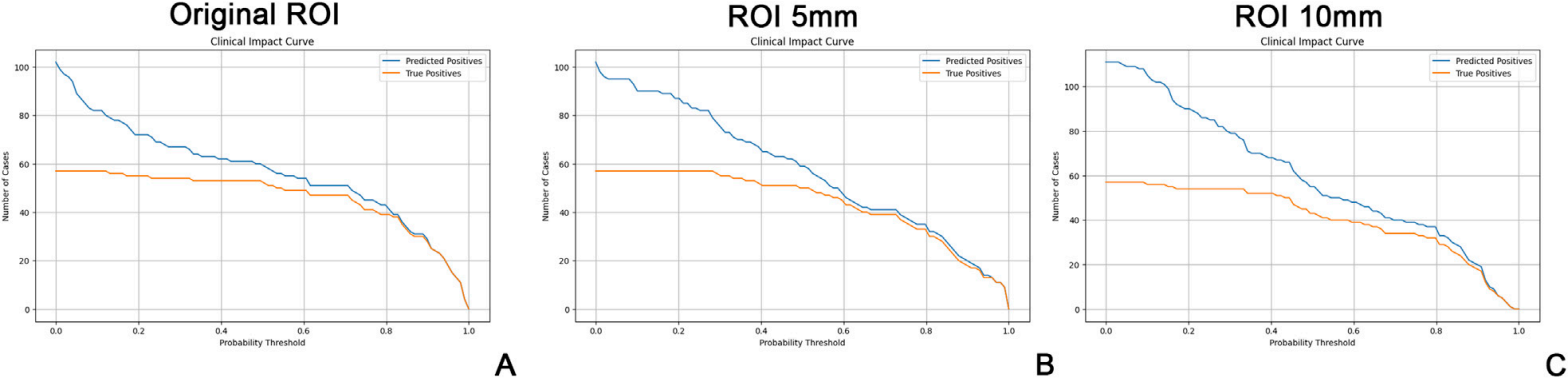
Comparison of cumulative incidence curves. **(A)** CIC of the original ROI. **(B)** CIC of the 5-mm expanded ROI. **(C)** CIC of the 10-mm expanded ROI.

**FIGURE 8:**
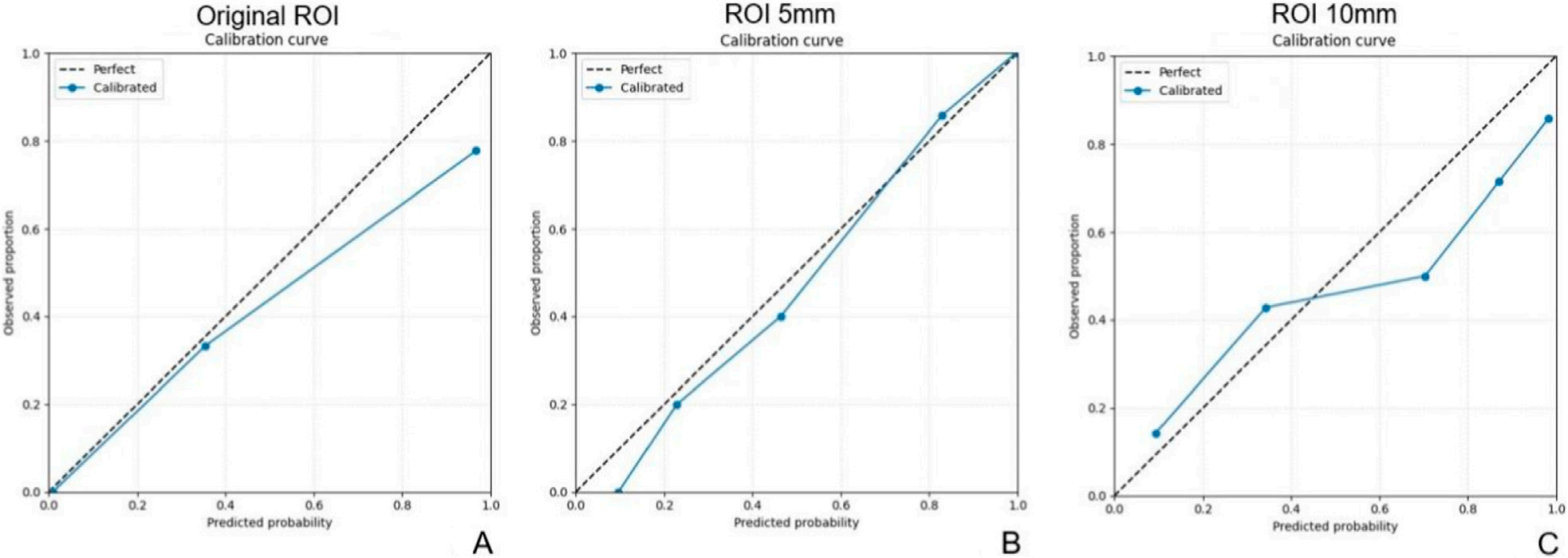
Comparison of the calibration curve. **(A)** CC of the original ROI. **(B)** CC of the 5 mm expanded ROI. **(C)** CC of the 10-mm expanded ROI.

CIC analysis (Figure 7) further supported these findings. The LR model with a 5 mm expanded ROI demonstrated superior clinical utility. When the high-risk threshold exceeded 68%, this model predicted a greater number of true positive cases compared with the observed prevalence, whereas the optimal benefit thresholds for the other models were >80%. Regarding calibration performance, the 5-mm expanded ROI model achieved the lowest Brier score (0.077), compared with the original ROI model (0.141) and the 10-mm expanded ROI (0.188), indicating better agreement between predicted and observed outcomes. The corresponding calibration curves are shown in Figure 8. Collectively, these results indicate that, in clinical application, the 5-mm expanded ROI model combined with LR provides superior diagnostic efficacy and calibration performance. This approach may facilitate more accurate assessment of treatment response and support more precise therapeutic decision-making.

## 4 Discussion

MRI is widely used for the diagnosis of chronic osteomyelitis (Massel et al., 2021; Iwasaki and Muraoka, 2020); however, limited evidence exists regarding its application in quantitative margin assessment and characterisation of perilesional tissue. Our previous studies demonstrated that an SVM model incorporating a 5-mm expansion beyond the original lesion improved diagnostic performance compared with the conventional ROI model (Jia et al., 2024). This observation suggested a potential strategy for more precise MRI-based diagnosis and highlighted the importance of optimal ROI selection. The present study sought to identify the most appropriate spatial range for radiomic feature extraction in osteomyelitis diagnosis. Three ROI configurations were constructed, as follows: the original osteomyelitis lesion ROI, a 5-mm expanded ROI and a 10-mm expanded ROI. These models were evaluated using 4 ML algorithms—KNN, SVM, RF and LR—to determine the most effective algorithm and ROI configuration. Among all models, LR achieved the highest AUC, accuracy and sensitivity. Notably, the 5 mm expanded ROI model demonstrated significantly superior diagnostic performance compared with the original ROI (DeLong test *p* < 0.05). This improvement may be anatomically explained by the inclusion of perilesional inflammatory or reactive changes beyond the visually defined lesion. The 5-mm expansion likely captured subtle pathological alterations in adjacent tissue that were not encompassed by the original ROI (DeLong test *p* < 0.05) (Wu et al., 2023; McNally et al., 2022; Lazzarini et al., 2002). Conversely, the 10-mm expanded ROI model exhibited significantly decreased AUC, sensitivity and accuracy (*p* < 0.05), likely due to the inclusion of excessive normal bone tissue, which introduced redundant information and reduced model development discriminative power. CIC analysis further indicated that the 5-mm expanded ROI model showed closer agreement between predicted and observed cases within the medium-risk threshold range, suggesting relatively more improved decision-curve behaviour. Nevertheless, these CIC findings remain exploratory and should not yet be interpreted as definitive clinical decision rules.

To minimise feature redundancy and improve model robustness, mRMR analysis was used, resulting in the selection of 10 representative features per model. Feature-weight analysis revealed distinct patterns across ROI configurations.

In the original ROI model, features such as 3Dfirstorder90Percentile (log-transformed), firstorderMedian (wavelet transform) and firstorderMinimum (first-order statistics) demonstrated high positive weights, whereas DependenceNonUniformity exhibited a high negative weight. The positive contribution of 3Dfirstorder90Percentile suggests that higher signal intensities were associated with osteomyelitis, consistent with increased MRI signal within inflamed bone marrow. The firstorderMinimum reflects low-intensity voxel values, capturing minimal signal regions. DependenceNonUniformity—often used to assess image texture uniformity (Çinarer et al., 2020)—showed a highly negative association, implying that the original ROI primarily comprises the lesion area, which appears as a highlighted region on MRI scans with consistent texture and elevated pixel values. The original ROI demonstrates a specific MRI scan pattern, where the lesion area is relatively uniform and conspicuous, facilitating its recognition by diagnosticians. However, from a diagnostic perspective, although the original ROI model accurately identifies chronic osteomyelitis, it may not sufficiently delineate potential comorbidities or guide surgical precision comprehensively.

With ROI expansion, additional texture heterogeneity was incorporated. In the 5 mm expanded model—the best-performing configuration—the maximum chromatic complexity feature showed a strong positive weight, reflecting increased textural heterogeneity characteristic of osteomyelitis (Su et al., 2023). The GLCM correlation feature demonstrated a negative weight, indicating reduced texture regularity (Bi et al., 2019). These findings imply that moderate expansion captures more complex and irregular perilesional patterns, corresponding to broader pathological involvement.

In the 10-mm expanded model, sphericity related to shape and HighGrayLevelEmphasis emerged as important features. Sphericity, which measures lesion roundness, showed a high negative weight, likely reflecting irregular lesion contours at wider margins (Priya et al., 2021). HighGrayLevelEmphasis, indicating dominance of higher grey-level intensities with limited variation, showed a high positive weight (Zhou et al., 2021). However, the inclusion of substantial healthy bone—typically exhibiting lower signal intensity—may have diluted lesion-specific information, explaining the observed decline in overall diagnostic performance. Although this study primarily focused on diagnostic modelling, the findings may provide indirect insights into surgical strategy. In orthopaedic clinical practice, debridement extending approximately 5 mm beyond the visible lesion is often considered the minimal margin to reduce recurrence risk, particularly when combined with antibiotic therapy. In more complex or uncertain cases, extending the margin to 10 mm may be considered to ensure comprehensive removal of potentially infected tissue. Our radiomic findings—indicating that the 5-mm zone contains concentrated pathological information, whereas the 10-mm zone introduces increased ‘healthy’ noise—statistically align with these clinical observations. Nonetheless, such interpretations should be approached cautiously.

The selection of 5- and 10-mm expansion distances was mainly based on MRI technical parameters, specifically slice thickness, to reduce partial volume effects and enhance feature stability. We acknowledge that these specific margins have not yet been confirmed by direct histopathological mapping in this study. Therefore, although these expansions may theoretically align with clinical debridement concepts, equating them to definitive ‘safe surgical margins’ remains speculative. Consequently, they should be currently interpreted as optimised zones for diagnostic feature extraction, with precise biological validation reserved for future prospective research.

Several limitations should be acknowledged. First, as a retrospective single-centre study, it is susceptible to selection and information bias. Previous research has indicated that clinical factors such as history of previous wounds (Lavery et al., 2009) and microbial infections (Bury et al., 2021) may influence the qualitative diagnosis of osteomyelitis. Second, external validation in an independent cohort was not performed, which may limit generalizability. Future investigations should incorporate multi-center datasets and integrate clinical variables with radiomic features to develop more comprehensive and clinically applicable predictive models.

## 5 Conclusion

This study proposes an innovative multi-level ROI expansion framework to enhance radiomics-based diagnosis and explore its relevance in chronic osteomyelitis. By comparing models constructed using the original ROI, a 5-mm expanded ROI and a 10-mm expanded ROI, we demonstrated that moderate expansion improves diagnostic performance, whereas excessive expansion may dilute lesion-specific information and reduce discriminative accuracy. Specifically, the combination of a 5-mm expanded ROI and the LR algorithm achieved the highest diagnostic efficacy, highlighting its potential clinical applicability and providing theoretical support for a strategy of appropriately moderate debridement expansion.

Although the 10-mm expanded ROI model showed slightly lower overall diagnostic performance than the 5-mm model, it incorporated a broader perilesional context and exhibited distinct value in characterising more extensive tissue alterations beyond the primary lesion boundary.

Collectively, these ROI expansion strategies offer a more comprehensive evaluation of lesion extent and provide data-driven support for precise and individualised surgical planning in chronic osteomyelitis.

## Data Availability

The original contributions presented in the study are included in the article/supplementary material, further inquiries can be directed to the corresponding authors.
