# Correction: Chronic osteomyelitis diagnosis using MRI: optimising radiomics models through multi-level region of interest extensions

**DOI:** 10.3389/fbioe.2026.1939949

**Published:** 2026-07-24

**Authors:** Hao Zheng, Qiyu Jia, Lin Jie, Jiaye Zhang, Shijie Jiang, Abudusalamu Alimujiang, Jian Guo, Junna Wang, Lanqi Fu, Zengru Xie, Chuang Ma

**Affiliations:** 1 Department of Radiology, The First Affiliated Hospital of Zhejiang Chinese Medical University (Zhejiang Provincial Hospital of Chinese Medicine), Hangzhou, China; 2 School of Medicine, Nankai University, Tianjin, China; 3 Department of Orthopedics, The First Affiliated Hospital of Xinjiang Medical University, Urumqi, China; 4 Xinjiang Clinical Research Center for Mental Health, The First Affiliated Hospital of Xinjiang Medical University, Xinjiang, China

**Keywords:** chronic osteomyelitis, machine learning, radiomics, region of interest, surgical decision-making

Authors Hao Zheng, Qiyu Jia, and Jie Lin were erroneously omitted as equal-contributing authors.

In the published article, there was an error in affiliation 2 for Qiyu Jia. The affiliation was incorrectly listed as “Department of Radiology, Nankai University School of Medicine, Tianjin, China.” The correct affiliation is “School of Medicine, Nankai University, Tianjin, China.”

The original version of this article has been updated.

